# 1204. Assessing Perceptions and Efficacy of COVID-19 Case and Contact Investigations – Dallas County, Texas, 2020

**DOI:** 10.1093/ofid/ofab466.1396

**Published:** 2021-12-04

**Authors:** Jose Serrano, Harper R Clouston, Jared Wiegand, Kyoo Shim, Nathan Popper, Kayla Maaraoui, Joshua Limsenben, Maxwell Hum, Robert Roseman, Ivorry Gomez, Wendy Chung

**Affiliations:** 1 Dallas County Health and Human Services, Dallas, Texas; 2 Dallas County Department of Health and Human Services, Dallas, TX

## Abstract

**Background:**

During 2020, a total of 193,318 cases of COVID-19 were reported in Dallas, with daily average case rates exceeding 50 per 100,000 for over 7 weeks. An adaptable survey functionality within a newly implemented COVID-19 surveillance system provided an opportunity to assess case knowledge and attitudes about isolation and contact tracing efforts.

**Methods:**

COVID-19 illnesses were classified using the 2020 CSTE case definitions. Cases were interviewed and records reviewed for exposures and illness characteristics. Supplemental questionnaires assessing knowledge of public health recommendations were given to a convenience sample of 987 cases during the month of December 2020. Fishers exact and chi-square analyses were performed using SAS 9.4.

**Results:**

Of the 987 respondents, 99% reported beginning isolation on or before receipt of test results, and 1% were not in isolation at the time of public health interview. Of cases reporting contacts, 92% had advised household members to quarantine prior to interview, and 91% did not want public health to call their household. Of cases reporting non-household close contacts, 75% had advised these contacts to quarantine prior to interview, and 91.3% did not want the health department to call these persons. Cases ≥ 65 years were less likely to have notified their own close contacts (OR: 0.2; 95% CI=0.1-0.8) of their test results, and more likely to prefer the health department to notify their household contacts of their positive result (OR: 4.1; 95% CI=1.3-12.5). Compared with White cases, Hispanic cases were less likely to be aware that their test was positive at the time of interview (OR: 0.3; 95% CI=0.1-0.7). Non-White cases were less likely to be aware of resources for food, rent and utility assistance prior to interview (OR: 0.25; 95% CI=0.1-0.7). All respondents perceived the public health interview to have been of some value to them, most often to answer their questions about retesting (51%) and duration of isolation (48%).

**Conclusion:**

The aversion of a majority of COVID-19 cases for health department notification of their contacts is a significant deterrent to name-based contact tracing approaches. Acknowledgement of this limitation could better focus existing resources on the delivery of expedited notifications and information to contacts by proxy.

**Disclosures:**

**All Authors**: No reported disclosures

